# The IPD and IMGT/HLA database: allele variant databases

**DOI:** 10.1093/nar/gku1161

**Published:** 2014-11-20

**Authors:** James Robinson, Jason A. Halliwell, James D. Hayhurst, Paul Flicek, Peter Parham, Steven G. E. Marsh

**Affiliations:** 1 Anthony Nolan Research Institute, Hampstead, London, NW3 2QG, UK; 2 UCL Cancer Institute, University College London, Hampstead, London, NW3 2QG, UK; 3 European Molecular Biology Laboratory, European Bioinformatics Institute, Wellcome Trust Genome Campus, Hinxton, Cambridge, CB10 1SD, UK; 4 Department of Structural Biology, Stanford University School of Medicine, Stanford, CA 94305-5136, USA

## Abstract

The Immuno Polymorphism Database (IPD) was developed to provide a centralized system for the study of polymorphism in genes of the immune system. Through the IPD project we have established a central platform for the curation and publication of locus-specific databases involved either directly or related to the function of the Major Histocompatibility Complex in a number of different species. We have collaborated with specialist groups or nomenclature committees that curate the individual sections before they are submitted to IPD for online publication. IPD consists of five core databases, with the IMGT/HLA Database as the primary database. Through the work of the various nomenclature committees, the HLA Informatics Group and in collaboration with the European Bioinformatics Institute we are able to provide public access to this data through the website http://www.ebi.ac.uk/ipd/. The IPD project continues to develop with new tools being added to address scientific developments, such as Next Generation Sequencing, and to address user feedback and requests. Regular updates to the website ensure that new and confirmatory sequences are dispersed to the immunogenetics community, and the wider research and clinical communities.

## INTRODUCTION

The Immuno Polymorphism Database (IPD) comprises a set of specialist databases related to the study of polymorphic genes in the immune system. Through the IPD project (1) we collaborate with specialist groups or nomenclature committees that provide and curate individual sections before they are submitted to IPD for online publication. The IPD project stores all the data in a set of related databases. IPD currently consists of five databases: IMGT/HLA, which contains sequences of the human Major Histocompatibility Complex (MHC); IPD-KIR, which contains the allelic sequences of the human Killer-cell Immunoglobulin-like Receptors; IPD-MHC, which is a database of sequences of the MHC of different species; IPD-HPA, which is a database of human platelet antigens; and IPD-ESTDAB, which provides access to the European Searchable Tumour Cell-Line Database, a cell bank of immunologically characterized melanoma cell lines.

## ALLELE DATABASES

A central function of the IPD project is as a database repository for the sequence data of polymorphic gene sequences. The project connects nomenclature committees for polymorphic gene systems with a core bioinformatics team who develop the tools and infrastructure required to maintain and publish the data. The aim of the project is to facilitate the work of the various nomenclature committees defining and curating the alleles within each gene system. Each allele is defined as a unique nucleotide sequence stored in the database and may cover the full-length of the gene, from UTR to UTR or just a mandatory number of exons. The entries in the database are not a description comparing two sequences. The submissions of a novel sequence must contain the sequence, and details of how this was obtained. A variant cannot not be reported as a Single Nucleotide Polymorphism (SNP) at a single position compared to a reference, without submitting proof of the sequence, the methodology used to obtain the sequence and details of the sample and its source. This ensures that the alleles seen in the databases are restricted to verified sequences and not a dataset of theoretical combinations of SNPs.

## THE IMGT/HLA DATABASE

**Figure 1.**
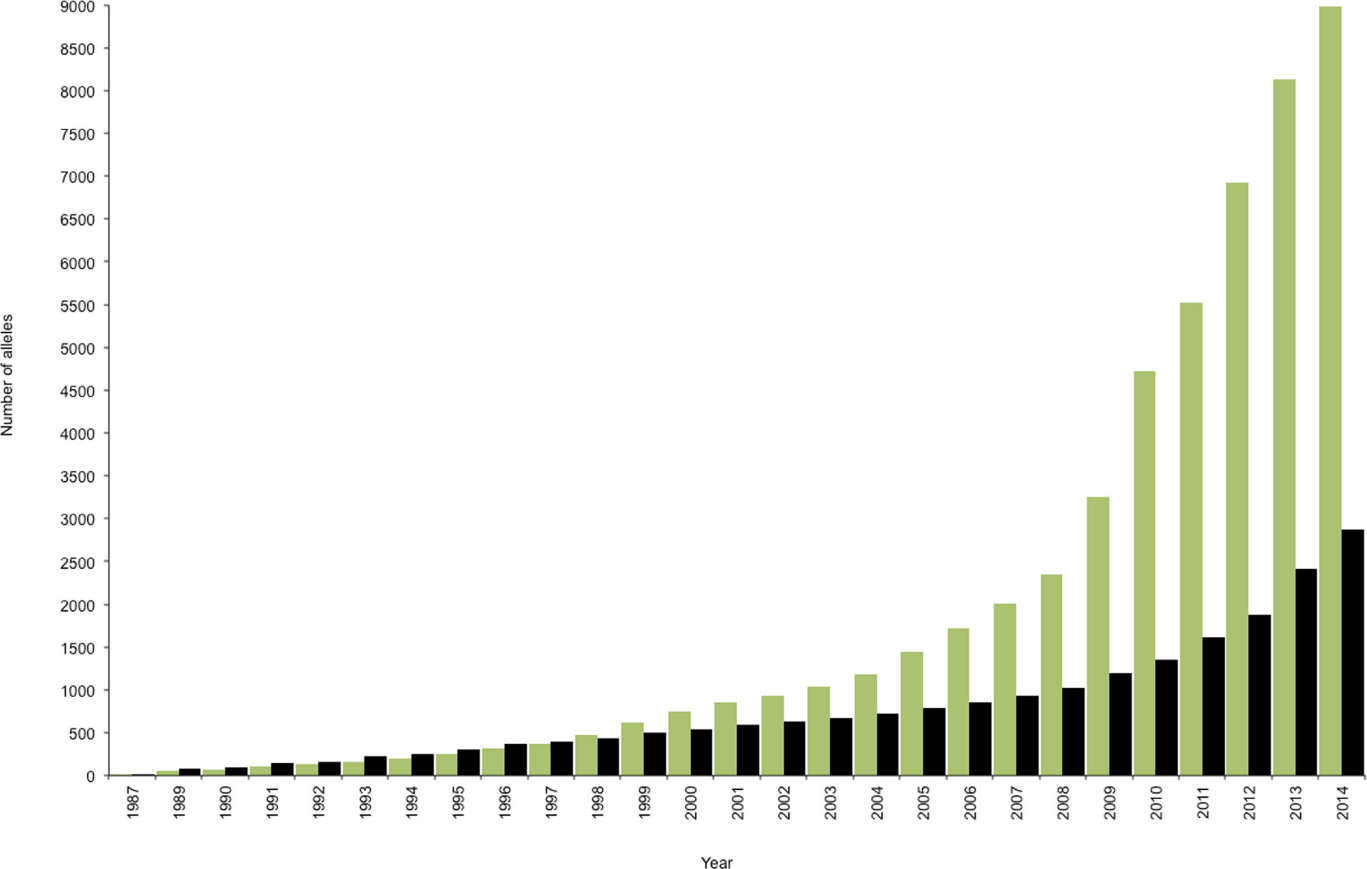
Growth of the IMGT/HLA Database. The number of allele sequences deposited annually in the IMGT/HLA Database is shown for class I (green), class II (black). The slope of the line reflects the rate of acquisition, which has accelerated in recent years.

The IMGT/HLA Database was established to provide a locus-specific database (LSDB) for the allelic sequences of the genes in the HLA system, also known as the human MHC. The IMGT/HLA Database was first released in 1998 and subsequently incorporated as a module of IPD in 2012. The MHC is one of the most complex and polymorphic regions of the human genome, with in excess of 220 genes (2). The core genes of interest in the HLA system are 21 polymorphic HLA genes, found within the 6p21.3 region of the short arm of human chromosome 6, whose protein products mediate human responses to infectious disease and influence the outcome of cell and organ transplants. The level of polymorphism seen in some of the genes is very high, with over 3000 variants seen in HLA-B; this level of variation can be considered hyper-polymorphic when compared to other gene systems. Three distinct regions have been identified within the MHC. The class I region is located at the telomeric end of the MHC and encodes the genes for the HLA class I molecules, HLA-A, -B and -C. These are co-dominantly expressed on the cell surface, and are responsible for presenting intracellularly derived peptides to CD8 positive T cells. The class II region lies at the centromeric end of the MHC and encodes HLA class II genes HLA-DRA, -DRB1, -DRB3, -DRB4, -DRB5, -DQA1, -DQB1, -DPA1 and -DPB1. HLA class II expression is limited to professional antigen presenting cells, where these molecules present extracellularly derived peptides to CD4 positive T cells. Located between the class I and class II regions lies the class III region where a number of non-HLA genes with immune function are located. With a nomenclature covering more than 50 genes and 12 000 alleles, there is an obvious need for a curated LSDB to manage these highly polymorphic variants. The first public release of the IMGT/HLA Database was made on the 16 December 1998 (3). Since then the database has been updated every three months, in a total of 64 releases, (Figure 1), to include all the publicly available sequences officially named by the WHO Nomenclature Committee at the time of release.

A major driving force behind the development and continued success of the IMGT/HLA Database is its use by the transplantation community. The HLA molecules play a key role in transplantation, with the success of kidney and bone marrow transplantation correlated with the degree to which donors and recipient are HLA matched. It has been shown that HLA matching is a critical determinant of outcome for patients receiving unrelated donor haematopoietic stem cells for haematological disorders (4). This has led to progressive improvements in the level of resolution achieved by HLA class I and II typing methods. HLA typing now focuses on distinguishing synonymous and non-synonymous differences within the nucleotide sequences that encode the protein domains of HLA class I and II molecules. These are the peptide binding domains that interact with variable lymphocyte receptors. The consequence of these improvements has required the development, for each polymorphic HLA class I and II gene, of a nucleotide sequence database that is both accurate and comprehensive. The use of a generalist sequence database for storing these sequences can be problematic due to inconsistencies in keyword descriptions, erroneous sequences and uncorrected errors.

The outcome and success of a transplant could be affected by a single SNP between the recipient and the donor. It is therefore vital for any repository storing HLA sequences to have high standards of both quality control and curation. To this end all sequences submitted to the IMGT/HLA Database are expected to meet a minimum set of agreed criteria. These criteria have been defined to ensure that both the quality of sequence submitted and the clinically relevant data are of the highest standard. Sequences that do not meet these standards are not accepted, although they may be found in other databases. Following initial checks on the data quality, the submissions are further checked to ensure that appropriate steps have been taken to correctly identify novel polymorphisms. The process uses in-house pipelines, which utilize Basic Local Alignment Search Tool (BLAST) (5) and Clustal (6), to both search and align the sequence submission, against both known and unnamed but submitted sequences, at the amino-acid, coding DNA sequence (CDS) and genomic level. The results of these searches highlight discrepancies against existing sequences, as well as providing detail analysis used in the provisional assignment of a name. In addition to automated analysis, curators analyse each sequence to ensure their validity and understand their mechanism of generation. Finally, no sequence is accepted into the database until it is formally named by experts within the field. The IMGT/HLA Database utilizes modern bioinformatics techniques to curate, annotate and analyse all submitted sequences, but in addition all sequences are checked by several experts at various stages of the submission process to ensure accuracy. To ensure the dissemination of these curated sequences to the wider community, once named, the alleles are added to the next nomenclature update. At this point, all named and publically available alleles are added to the public copy of the IMGT/HLA Database, the online resources are updated, and the wider community is notified of the release. At this point the users of the database are able to download the most recent version of the database to update their own local resources, ensuring that clinical testing is performed on the most recent data available.

## HLA POLYMORPHISM AND NEXT GENERATION SEQUENCING

In recent years there has been a demand to increase the accuracy and length of sequences, while where possible lowering the cost of sequencing. This has led to the development of ‘Next Generation Sequencing’ (NGS) techniques which have enabled affordable, routine high-throughput sequencing approaches (6–10). High-throughput sequencing generates billions of sequence bases through parallel sequencing such that the same molecule(s) is sequenced multiple times in the same experiment leading, in turn, to vast amounts of new data being made available. Within immunogenetics, this has led to new approaches to sequencing the HLA genes, potentially providing greater accuracy and coverage. These developments have far-reaching implications for both immunogenetics research and clinical applications of Haematopoietic Stem Cell Transplantation (HSCT). HSCT uses DNA sequences to identify potential transplant donors with patients, as HLA matching is a critical factor when considering potential donors for patients receiving allogeneic transplants for haematological disorders (4,7).

**Figure 2.**
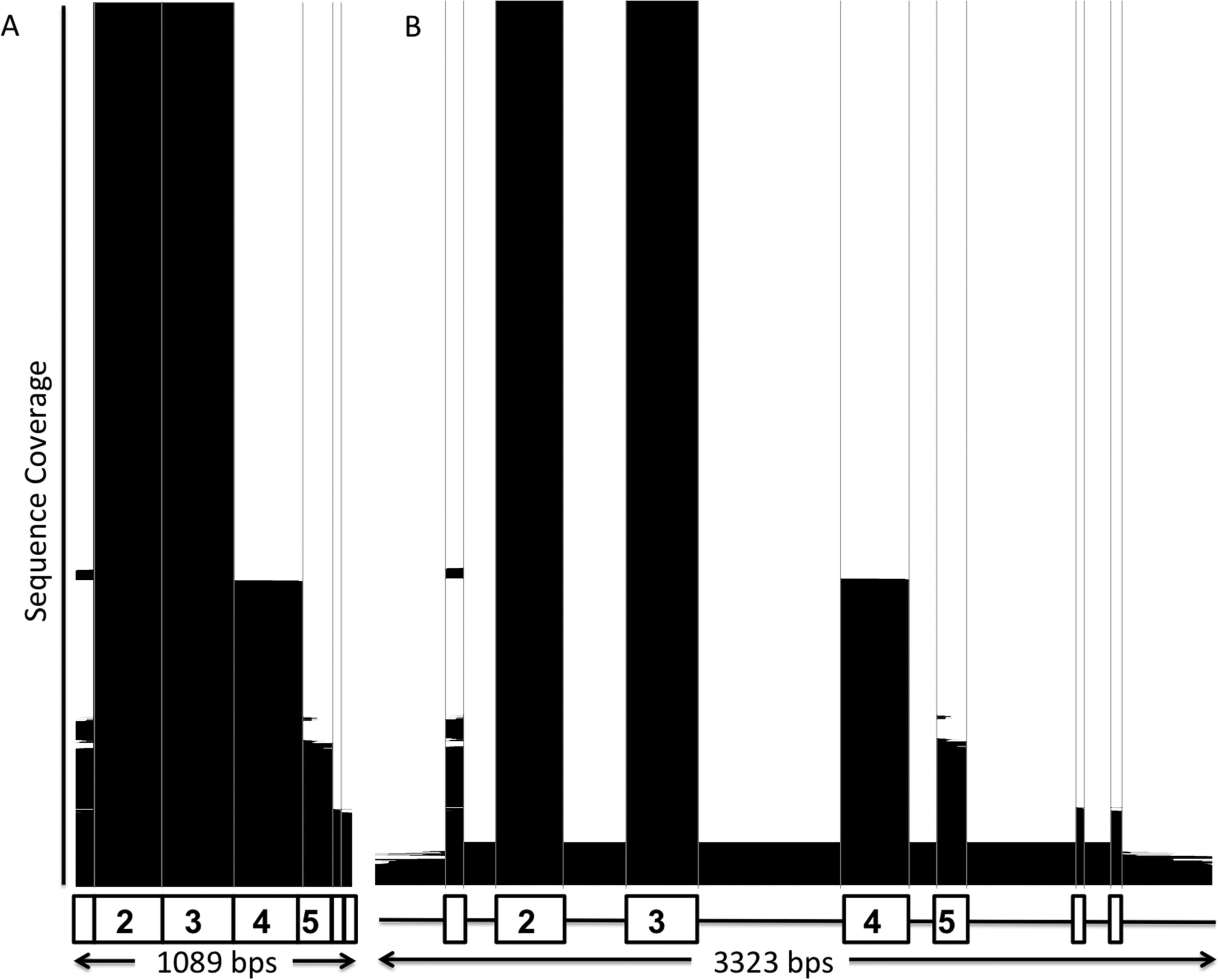
Sequence Coverage of HLA-B in the IMGT/HLA Database. Panel (**A**) represent the HLA-B CDS sequences in the database, Panel (**B**) represents the HLA-B gDNA sequences. The white areas represent the unsequenced regions. The black areas represent the sequenced regions. The sequences are ordered by the length of sequence covered, the plots clearly show the exon 2 and 3 regions which are mandatory requirements for submission to the IMGT/HLA Database.

These recent developments in NGS methods have seen the user base of the IMGT/HLA Database expand with the additional interest in the highly curated datasets that are needed for analysis of data produced using the next generation of technology. The IMGT/HLA Database has historically been populated with data produced by a number of techniques that have focussed specifically on the more variable regions of the HLA molecule, specifically exons 2 and 3 of class I and exon 2 of class II. This has meant that whilst the database holds a large number of polymorphic sequences, these sequences can be limited to particular regions of the DNA. Figure 2 shows two coverage plots for HLA-B, the most polymorphic locus, detailing the amino-acid sequence, the CDS and the genomic DNA (gDNA) sequence. The concentration of coverage around exons 2 and 3 can be clearly seen, particularly as sequencing of these exons has been the minimum requirement for acceptance. The coverage of the flanking exons is much lower with <25% of the sequences containing data on exon 1 and <35% containing data on exon 4. The average coverage for gDNA is <10% of the alleles at a given gene.

The generation of long-read length sequences will see an increased number of sequences deposited into the database. Whilst some of these sequences will extend existing entries, and fill out missing sequence, it is expected that many of the entries will provide novel sequences. The impact of this deluge of data in the clinical setting and its utility has yet to be measured. Under current practices, the influx of genomic data will have little effect on matching algorithms that are based solely on exon 2 and exon 3 sequence for class I or the exon 2 sequence for class II. However, with this increase in data, comes the need to analyse the impact of polymorphisms outside of these regions and assess these positions for their clinical relevance. While the expected influx of sequences also raises questions regarding the suitability of both the database and the nomenclature for the task, the HLA nomenclature was updated in 2010 (8), and the changes implemented should ensure that even with a tsunami of new sequences, the nomenclature is fit for purpose. The underlying infrastructure used to maintain the IMGT/HLA Database is currently been reviewed and new tools and analysis pipelines are being developed to assist in the curation of sequences generated by NGS techniques.

The analysis of the data produced by NGS techniques, whether it is the long reads associated with the Pacific Biosciences SMRT technology (9,10) or shorter tiled reads associated with Roche 454 (11), Illumina (12) and ION-PGM, Ion-Torrent (13), still require an accurate reference database in order to assess the quality of the sequences produced. The hyper-polymorphic nature of HLA means that it can be difficult to accurately phase and implement sequencing analysis when a reference sequence is either unavailable or highly variable. The IMGT/HLA Database is currently focusing on addressing the challenge posed by this lack of reference data. With over 12 000 known alleles, it may not be possible to source material for all of these to obtain the full genomic sequence. Instead, the expected influx of NGS data will begin to populate the missing areas of the database. The IMGT/HLA Database will currently only accept full-length genomic sequences where the phasing of all of the tiled reads can be proven. The concern is that without accurate phasing the inferred full-length sequences produced by assembling fragments may be inaccurate. Assemblies produced based on regions that miss information or with low coverage in the reference database, may be of low accuracy or incorrectly phased. Due to the hyper-polymorphic nature of the key genes sequenced for HSCT, SNPs that may be key to assembling full-length sequences may not be available from the database, suggesting it is not just coverage but depth of coverage that is essential for accurate assembly. The IMGT/HLA Database contains genomic sequences for all the main serological groups for class I (HLA-A, -B and -C) but the coverage for class II is much lower, which may effect the accuracy of any assembled sequence.

**Figure 3.**
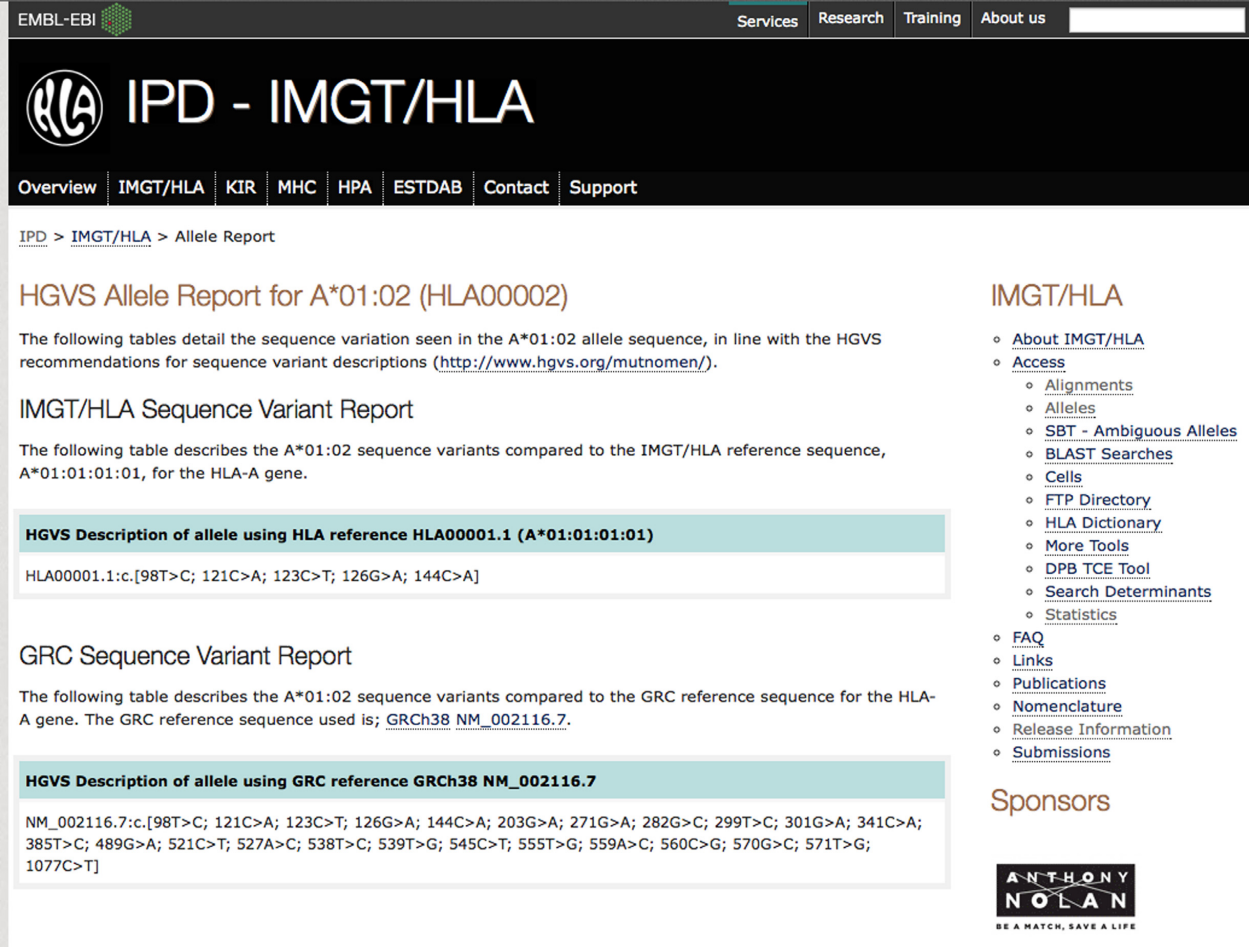
IMGT/HLA HGVS Variant Report. The figure shows an example of an allele report, which utilizes the HGVS variant reporting format to describe the allele rather than display the entire sequence. The variations are described in relation to the WHO HLA Reference Sequence and a GRC reference sequence.

## ALTERNATIVE DESCRIPTIONS FOR HLA ALLELE VARIATION

The IMGT/HLA Database displays allelic variation against a reference sequence for each gene. The differences can be seen visually in the sequence alignment tool. The Human Genome Variation Society (HGVS) reporting system (14,15), is another method for reporting alleles. The HGVS description compares each allele to a reference sequence and describes the change, rather than providing separate sequences for both alleles. In 2014, the IMGT/HLA Database has introduced HGVS reporting for all alleles, as part of the allele report page on the main website, see Figure 3. This report lists the variation seen in each allele against both the WHO Nomenclature Committee for Factors of the HLA System approved Reference Sequence, and the GRC Reference Sequence (GRCh38/hg38) (16). It should be noted that these references often refer to different sequences, and for some genes the GRC reference sequences do not match existing sequences within the IMGT/HLA Database. The hyper-polymorphic nature of the HLA system is emphasized when using the HGVS reporting methods. HLA-B currently has over 3500 known variant sequences that differ by at least a SNP within the gDNA sequence. The HGVS report for HLA-B needs to list over 113 629 descriptors to cover this polymorphism just at the CDS level. Using an alternative reference sequence, B*15:01:01:01 compared to B*07:02:01, reduces the number of descriptors by 23%. This suggests that the HGVS descriptors are less reliable indices of the levels of variation as they can be easily influenced by the choice of reference sequence. Further development of these descriptors, their use, and publication will allow for linking the descriptors catalogued in the IMGT/HLA Database to other reference databases. This is of particular interest for NGS analysis, for example, the establishment of cross references between the IMGT/HLA HGVS descriptors and the rs# used in dbSNP (17), which is an increasingly common request from the new users of the database.

## TOOLS AVAILABLE AT IMGT/HLA

The IMGT/HLA Database provides a diversity of tools for the analysis of HLA sequences. Some of these tools were custom written for the IMGT/HLA Database, whereas others were incorporated from the existing set of tools described on the EBI website (18,19). The website includes tools for producing user-defined sequence alignments at the protein, cDNA and gDNA level. The user is also able to perform queries for specific HLA alleles; the output provides access to detailed information on any HLA allele, including information on the ethnic origin of the source material, database cross-references and seminal publications. This information is also available through integration with European Bioinformatic Institute (EBI) EB-Eye search engine (20).

Sequence data, both nucleotide and protein, from IPD is incorporated into the EBI suite of search tools including the FASTA suite of programmes (21) and the BLAST (5) and are downloadable from the EBI's File Transfer Protocol (FTP) directory in a variety of commonly used formats such as FASTA, MSF and PIR.

## IMGT/HLA AS A MODEL FOR OTHER HIGHLY POLYMORPHIC GENE SYSTEMS

The HLA Nomenclature and its publication through the IMGT/HLA Database has been taken as a model by other groups working on curating MHC sequence variation. The MHC sequences of many different species have been reported (22–33), often in different formats and with different nomenclature systems used in the naming and identification of new genes and alleles in each species (34). This disparate approach has led to many individual studies, often unrelated, with the potential for conflicting nomenclature. The nomenclature for MHC genes and alleles in species other than humans (1,8) and mice (35,36) has historically been overseen either informally by groups generating sequences, or by formal nomenclature committees set up by the International Society for Animal Genetics (ISAG) (37). This work is now overseen by the Comparative MHC Nomenclature Committee and is supported by ISAG and the Veterinary Immunology Committee (VIC) of the International Union of Immunological Societies (IUIS) (38). With the high degree of similarity seen in the sequences of the MHC from a number of different species (39) a consistent methodology for the curation, naming and publication of these sequences is recommended. By bringing the work of different nomenclature committees together a central resource that facilitates further research on the MHC of each can be developed (40). The first version of the IPD-MHC database involved the work of groups specializing in non-human primates (NHP) (32), canines (DLA) (28) and felines (FLA) (41) and incorporated all data previously available in the IMGT/MHC Database (40). Further developments have been able to add sequences from cattle (BoLA) (33), teleost fish (42), rats (RT1) (43), sheep (OLA) (31) and swine (SLA) (30). In 2012 the nomenclature used to describe the alleles of non-human primates was extensively revised and updated (32). This was accompanied by updating the NHP section of IPD-MHC, which currently contains over 4000 alleles covering 47 species of apes, old world and new world monkeys. The management of the sequences within IPD-MHC and the provision of an online submission tool has enabled these databases to grow. The number of sequences deposited in IPD-MHC has increased by at least 10% each year. This has resulted in regular publications reporting updates or changes to the nomenclature (31–33,44).

The IMGT/HLA Database model can also be applied outside the MHC, as is seen in the IPD-KIR Database. The KIR genes are members of the immunoglobulin super family (IgSF) formerly called Killer-cell Inhibitory Receptors. KIRs are highly polymorphic both at the allelic and haplotypic levels (45). They are composed of two or three Ig-domains, a transmembrane region and cytoplasmic tail, which can in turn be short (activatory) or long (inhibitory). Due to the complexity in the KIR region and KIR sequences, a KIR Nomenclature Committee was established in 2002 to undertake the naming of human KIR allele sequences. The first KIR Nomenclature report was published in 2003 (46), which coincided with the first release of the IPD-KIR database. The number of officially named human KIR alleles has increased since the initial release, which contained 89 alleles. As of January 2014, there are now over 600 alleles, which code for over 320 unique protein sequences. Table 1 provides further information on the content of the various projects.

**Table 1.** 

Project	Description	Species	Genes	Sequences
IPD-IMGT/HLA	Human major histocompatibility complex and related genes	1	38	12 406
IPD-MHC	Non-human major histocompatibility complex	57	384	5669
IPD-KIR	Human Killer-cell Immunoglobulin-like Receptors	1	16	678
IPD-HPA	Human Platelet Antigens	1	6	22
IPD-ESTDAB	The European Searchable Tumour Line Database (ESTDAB) Database and Cell Bank contains 211 cells characterized for 240 markers	1	NA	NA

## IPD-HPA AND IPD-ESTAB

The IPD-HPA and IPD-ESTDAB projects are housed within IPD but do not share the same structure and tools as the other projects. The IPD-HPA Database provides a centralized repository for the data, which define the human platelet antigens (HPA). Alloantibodies against human platelet antigens are involved in neonatal alloimmune thrombocytopenia, post-transfusion purpura and refractoriness to random donor platelets. The HPA nomenclature system was adopted in 1990 (47) to overcome problems with the previous nomenclature. Since then, more antigens have been described and the molecular basis of many have been resolved, and the nomenclature was revised in 2003 (48). The European Searchable Tumour Line Database (ESTDAB) Database and Cell Bank (49,50) provide a service enabling investigators to search online for HLA typed immunologically characterized tumour cells as part of the European Commission Fifth Framework Infrastructures Program.

## FUTURE DEVELOPMENTS

A major challenge for the developers and the curators of the databases is to keep up with the increasing number of allele sequences that are being submitted. In recent years the number of sequences in the database increased on average by 29% each year. The database must develop new tools for the visualization of sequences whilst maintaining the high standards set in the presentation and quality of the HLA sequences and nomenclature to the research community. The techniques behind NGS offer the potential to phase polymorphisms across genes, rather than within the individual genes. The database will need to consider how this type of data can be made available and whether a nomenclature for these haplotypes needs implementing or whether existing reporting formats (51) can be utilized to present this data, using the HLA Nomenclature and allele designations as core components in reporting these new variants. The database aims to continually develop new tools and refine existing tools to meet this challenge. These challenges must be met by all IPD projects.

## CONCLUSIONS

The IPD provides a centralized resource for everybody interested, clinically or scientifically, in the MHC system. The database and accompanying tools allow the study of these alleles from a single site on the World Wide Web. It aids in the management and development of nomenclature, providing a continuing and updated resource for the Nomenclature Committees. The challenges for the database are to keep up with the increase in submitted sequences, keep pace with the increasing difficulties in performing analyses on larger datasets and develop new tools for the visualization of sequences whilst maintaining the high standards set in the presentation and quality of the sequences and nomenclature to the research community.

## LICENSING

The IMGT/HLA database is covered by the Creative Commons Attribution-NoDerivs Licence, which is applicable to all copyrightable parts of the database, which includes the sequence alignments. This means that users are free to copy, distribute, display and make commercial use of the databases in all jurisdictions provided they give the appropriate credit (52,53). Support for the database is requested from commercial users of the database resources. If users intend to distribute a modified version of the data in any form, then they must ask for permission; this can be done by contacting hla@alleles.org for further details of how modified data can be reproduced.

## AVAILABILITY

IPD Homepage: http://www.ebi.ac.uk/ipd/
IPD FTP Site: ftp://ftp.ebi.ac.uk/pub/databases/ipd/
IMGT/HLA Homepage: http://www.ebi.ac.uk/ipd/imgt/hla/
IMGT/HLA FTP Site: ftp://ftp.ebi.ac.uk/pub/databases/ipd/imgt/hla/
Contact: hla@alleles.org

## References

[1] Robinson J., Halliwell J.A., McWilliam H., Lopez R., Parham P., Marsh S.G.E. (2013). The IMGT/HLA Database. Nucleic Acids Res..

[2] Horton R., Wilming L., Rand V., Lovering R.C., Bruford E.A., Khodiyar V.K., Lush M.J., Povey S., Talbot C.C. Jr., Wright M.W. (2004). Gene map of the extended human MHC. Nat Rev Genet..

[3] Robinson J., Bodmer J.G., Malik A., Marsh S.G.E. (1998). Development of the international immunogenetics HLA database. Hum. Immunol..

[4] Shaw B.E., Mayor N.P., Russell N.H., Apperley J.F., Clark R.E., Cornish J., Darbyshire P., Ethell M.E., Goldman J.M., Little A.M. (2010). Diverging effects of HLA-DPB1 matching status on outcome following unrelated donor transplantation depending on disease stage and the degree of matching for other HLA alleles. Leukemia.

[5] Altschul S.F., Gish W., Miller W., Myers E.W., Lipman D.J. (1990). Basic local alignment search tool. J. Mol. Biol..

[6] Thompson J.D., Higgins D.G., Gibson T.J. (1994). CLUSTAL W: improving the sensitivity of progressive multiple sequence alignment through sequence weighting, position-specific gap penalties and weight matrix choice. Nucleic Acids Res..

[7] Lee S.J., Klein J., Haagenson M., Baxter-Lowe L.A., Confer D.L., Eapen M., Fernandez-Vina M., Flomenberg N., Horowitz M., Hurley C.K. (2007). High-resolution donor-recipient HLA matching contributes to the success of unrelated donor marrow transplantation. Blood.

[8] Marsh S.G.E., Albert E.D., Bodmer W.F., Bontrop R.E., Dupont B., Erlich H.A., Fernandez-Vina M., Geraghty D.E., Holdsworth R., Hurley C.K. (2010). Nomenclature for factors of the HLA system, 2010. Tissue Antigens.

[9] Korlach J., Bjornson K.P., Chaudhuri B.P., Cicero R.L., Flusberg B.A., Gray J.J., Holden D., Saxena R., Wegener J., Turner S.W. (2010). Real-time DNA sequencing from single polymerase molecules. Methods Enzymol..

[10] Eid J., Fehr A., Gray J., Luong K., Lyle J., Otto G., Peluso P., Rank D., Baybayan P., Bettman B. (2009). Real-time DNA sequencing from single polymerase molecules. Science.

[11] Moonsamy P.V., Williams T., Bonella P., Holcomb C.L., Hoglund B.N., Hillman G., Goodridge D., Turenchalk G.S., Blake L.A., Daigle D.A. (2013). High throughput HLA genotyping using 454 sequencing and the Fluidigm Access Array System for simplified amplicon library preparation. Tissue Antigens.

[12] Lange V., Bohme I., Hofmann J., Lang K., Sauter J., Schone B., Paul P., Albrecht V., Andreas J.M., Baier D.M. (2014). Cost-efficient high-throughput HLA typing by MiSeq amplicon sequencing. BMC Genomics.

[13] De Santis D., Dinauer D., Duke J., Erlich H.A., Holcomb C.L., Lind C., Mackiewicz K., Monos D., Moudgil A., Norman P. (2013). 16(th) IHIW : review of HLA typing by NGS. Int. J. Immunogenet..

[14] den Dunnen J.T., Antonarakis S.E. (2000). Mutation nomenclature extensions and suggestions to describe complex mutations: a discussion. Hum. Mutat..

[15] Taschner P.E., den Dunnen J.T. (2011). Describing structural changes by extending HGVS sequence variation nomenclature. Hum. Mutat..

[16] Church D.M., Schneider V.A., Graves T., Auger K., Cunningham F., Bouk N., Chen H.C., Agarwala R., McLaren W.M., Ritchie G.R. (2011). Modernizing reference genome assemblies. PLoS Biol..

[17] Sherry S.T., Ward M.H., Kholodov M., Baker J., Phan L., Smigielski E.M., Sirotkin K. (2001). dbSNP: the NCBI database of genetic variation. Nucleic Acids Res..

[18] Goujon M., McWilliam H., Li W., Valentin F., Squizzato S., Paern J., Lopez R. (2010). A new bioinformatics analysis tools framework at EMBL-EBI. Nucleic Acids Res..

[19] McWilliam H., Valentin F., Goujon M., Li W., Narayanasamy M., Martin J., Miyar T., Lopez R. (2009). Web services at the European Bioinformatics Institute-2009. Nucleic Acids Res..

[20] Valentin F., Squizzato S., Goujon M., McWilliam H., Paern J., Lopez R. (2010). Fast and efficient searching of biological data resources–using EB-eye. Brief. Bioinform..

[21] Pearson W.R., Lipman D.J. (1988). Improved tools for biological sequence comparison. Proc. Natl. Acad. Sci. U.S.A..

[22] Longenecker B.M., Mosmann T.R. (1981). Nomenclature for chicken MHC (B) antigens defined by monoclonal antibodies. Immunogenetics.

[23] Briles W.E., Bumstead N., Ewert D.L., Gilmour D.G., Gogusev J., Hala K., Koch C., Longenecker B.M., Nordskog A.W., Pink J.R. (1982). Nomenclature for chicken major histocompatibility (B) complex. Immunogenetics.

[24] (1991). Leukocyte antigens in cattle, sheep and goats. Nomenclature. Vet. Immunol. Immunopathol..

[25] Davies C.J., Andersson L., Joosten I., Mariani P., Gasbarre L.C., Hensen E.J. (1992). Characterization of bovine MHC class II polymorphism using three typing methods: serology, RFLP and IEF. Eur. J. Immunogenet..

[26] Naessens J. (1993). Leukocyte antigens of cattle and sheep. Nomenclature. Vet. Immunol. Immunopathol..

[27] Kennedy L.J., Altet L., Angles J.M., Barnes A., Carter S.D., Francino O., Gerlach J.A., Happ G.M., Ollier W.E., Polvi A. (2000). Nomenclature for factors of the dog major histocompatibility system (DLA), 1998: first report of the ISAG DLA Nomenclature Committee. Anim. Genet..

[28] Kennedy L.J., Angles J.M., Barnes A., Carter S.D., Francino O., Gerlach J.A., Happ G.M., Ollier W.E., Thomson W., Wagner J.L. (2001). Nomenclature for factors of the dog major histocompatibility system (DLA), 2000: second report of the ISAG DLA Nomenclature Committee. Anim. Genet..

[29] Miller M.M., Bacon L.D., Hala K., Hunt H.D., Ewald S.J., Kaufman J., Zoorob R., Briles W.E. (2004). Nomenclature for the chicken major histocompatibility (B and Y) complex. Immunogenetics.

[30] Smith D.M., Lunney J.K., Ho C.S., Martens G.W., Ando A., Lee J.H., Schook L., Renard C., Chardon P. (2005). Nomenclature for factors of the swine leukocyte antigen class II system, 2005. Tissue Antigens.

[31] Ballingall K.T., Herrmann-Hoesing L., Robinson J., Marsh S.G.E., Stear M.J. (2011). A single nomenclature and associated database for alleles at the major histocompatibility complex class II DRB1 locus of sheep. Tissue Antigens.

[32] de Groot N.G., Otting N., Robinson J., Blancher A., Lafont B.A., Marsh S.G.E., O'Connor D.H., Shiina T., Walter L., Watkins D.I. (2012). Nomenclature report on the major histocompatibility complex genes and alleles of Great Ape, Old and New World monkey species. Immunogenetics.

[33] Hammond J.A., Marsh S.G.E., Robinson J., Davies C.J., Stear M.J., Ellis S.A. (2012). Cattle MHC nomenclature: is it possible to assign sequences to discrete class I genes. Immunogenetics.

[34] Klein J., Bontrop R.E., Dawkins R.L., Erlich H.A., Gyllensten U.B., Heise E.R., Jones P.P., Parham P., Wakeland E.K., Watkins D.I. (1990). Nomenclature for the major histocompatibility complexes of different species: a proposal. Immunogenetics.

[35] Rodgers J.R., Levitt J.M., Cresswell P., Lindahl K.F., Mathis D., Monaco J.T., Singer D.S., Ploegh H.L., Bryant P.W. (1999). A nomenclature solution to mouse MHC confusion. J. Immunol..

[36] Eppig J.T., Blake J.A., Bult C.J., Kadin J.A., Richardson J.E. (2012). The Mouse Genome Database (MGD): comprehensive resource for genetics and genomics of the laboratory mouse. Nucleic Acids Res..

[37] Ellis S.A., Bontrop R.E., Antczak D.F., Ballingall K., Davies C.J., Kaufman J., Kennedy L.J., Robinson J., Smith D.M., Stear M.J. (2006). ISAG/IUIS-VIC Comparative MHC Nomenclature Committee report, 2005. Immunogenetics.

[38] Ballingall K.T. (2012). Progress of the comparative MHC Committee and a summary of the comparative MHC Workshops held at the 32nd ISAG, Edinburgh and the 9th IVIS, Tokyo, 2010. Vet. Immunol. Immunopathol..

[39] Parham P. (1999). Virtual reality in the MHC. Immunol. Rev..

[40] Robinson J., Waller M.J., Parham P., de Groot N., Bontrop R., Kennedy L.J., Stoehr P., Marsh S.G.E. (2003). IMGT/HLA and IMGT/MHC: sequence databases for the study of the major histocompatibility complex. Nucleic Acids Res..

[41] Drake G.J., Kennedy L.J., Auty H.K., Ryvar R., Ollier W.E., Kitchener A.C., Freeman A.R., Radford A.D. (2004). The use of reference strand-mediated conformational analysis for the study of cheetah (Acinonyx jubatus) feline leucocyte antigen class II DRB polymorphisms. Mol. Ecol..

[42] Lukacs M.F., Harstad H., Bakke H.G., Beetz-Sargent M., McKinnel L., Lubieniecki K.P., Koop B.F., Grimholt U. (2010). Comprehensive analysis of MHC class I genes from the U-, S-, and Z-lineages in Atlantic salmon. BMC Genomics.

[43] Fujii H., Kakinuma M., Yoshiki T., Natori T. (1991). Polymorphism of the class II gene of rat major histocompatibility complex, RT1: partial sequence comparison of the first domain of the RT1.B beta 1 alleles. Immunogenetics.

[44] Ho C.S., Lunney J.K., Ando A., Rogel-Gaillard C., Lee J.H., Schook L.B., Smith D.M. (2009). Nomenclature for factors of the SLA system, update 2008. Tissue Antigens.

[45] Garcia C.A., Robinson J., Guethlein L.A., Parham P., Madrigal J.A., Marsh S.G.E. (2003). Human KIR sequences 2003. Immunogenetics.

[46] Marsh S.G.E., Parham P., Dupont B., Geraghty D.E., Trowsdale J., Middleton D., Vilches C., Carrington M., Witt C., Guethlein L.A. (2003). Killer-cell immunoglobulin-like receptor (KIR) nomenclature report, 2002. Tissue Antigens.

[47] von dem Borne A.E., Decary F. (1990). Nomenclature of platelet-specific antigens. Hum. Immunol..

[48] Metcalfe P., Watkins N.A., Ouwehand W.H., Kaplan C., Newman P., Kekomaki R., DeHaas M., Aster R., Shibata Y., Smith J. (2003). Nomenclature of human platelet antigens. Vox Sang..

[49] Pawelec G., Marsh S.G.E. (2006). ESTDAB: a collection of immunologically characterised melanoma cell lines and searchable databank. Cancer Immunol. Immunother..

[50] Robinson J., Roberts C.H., Dodi I.A., Madrigal J.A., Pawelec G., Wedel L., Marsh S.G. (2009). The European searchable tumour line database. Cancer Immunol. Immunother..

[51] Milius R.P., Mack S.J., Hollenbach J.A., Pollack J., Heuer M.L., Gragert L., Spellman S., Guethlein L.A., Trachtenberg E.A., Cooley S. (2013). Genotype List String: a grammar for describing HLA and KIR genotyping results in a text string. Tissue Antigens.

[52] Robinson J., Malik A., Parham P., Bodmer J.G., Marsh S.G.E. (2000). IMGT/HLA database–a sequence database for the human major histocompatibility complex. Tissue Antigens.

[53] Robinson J., Waller M.J., Fail S.C., McWilliam H., Lopez R., Parham P., Marsh S.G.E. (2009). The IMGT/HLA database. Nucleic Acids Res..

